# NF-kappaB inducing kinase (NIK) is a key regulator of inflammation-induced angiogenesis

**DOI:** 10.1186/1479-5876-10-S3-O1

**Published:** 2012-11-28

**Authors:** Ae-Ri Noort, Katinka PM van Zoest, Pieter Koolwijk, Paul-Peter Tak, Sander W Tas

**Affiliations:** 1 Division of Clinical Immunology & Rheumatology, Academic Medical Center, University of Amsterdam, The Netherlands; 2 Dept. of Physiology, Institute for Cardiovascular Research (ICaR-VU), VU University Medical Center, Amsterdam, The Netherlands

## Background

In rheumatoid arthritis (RA) synovial tissue (ST) angiogenesis can be observed already in the earliest phase of disease. The chemokine CXCL12, which is induced via the non-canonical nuclear factor-kappaB (NF-kB) pathway, plays an important role in angiogenesis, lymphocyte transendothelial migration, and the homing of endothelial progenitor cells. Therefore, the non-canonical pathway, with its key mediator NF-kB inducing kinase (NIK), may play an important role in pathological angiogenesis and the perpetuation of synovial inflammation in RA.

## Objective

To study the role of non-canonical NF-kB signaling in pathological angiogenesis in RA.

## Methods

Expression of NIK and CXCL12 in RA ST was evaluated using immunofluorescence microscopy (IF). Angiogenesis was studied in endothelial cells (EC) in vitro using the tube formation assay and ex vivo by comparing WT and NIK^-/-^ mice in the aortic ring assay. Physiological (developmental) angiogenesis was evaluated by isolectin B4 staining of the retina followed by confocal microscopy. The contribution of NIK to synovial angiogenesis was studied in vivo in antigen-induced arthritis (AIA).

## Results

NIK, p52 and CXCL12 were highly expressed in EC in RA ST, mainly in small (newly formed) blood vessels. Stimuli that induce non-canonical NF-kB signaling (lymphotoxin (LT), LIGHT, and CD40L) significantly enhanced in vitro tube formation 2,5-fold (p<0.05), which could be completely blocked by siRNA targeting NIK or IKKα. Aortic rings from WT and NIK^-/-^ mice showed normal TNF- and VEGF-induced microvessel outgrowth. In contrast, whereas non-canonical NF-kB stimuli induced microvessel outgrowth in WT mice (unstim 29.94 ± 6.08 vs. LT 159.1 ± 50.24 vs. LIGHT 110.3 ± 17.68 (mm^2) p<0.05), no microvessel outgrowth was observed in aortic rings from NIK^-/-^ mice (unstim 28.74 ± 15.89 vs. LT 45.9 ± 16.71 vs. LIGHT 43.41 ± 15.73 (mm^2)). In line with this, NIK^-/-^ mice exhibited normal developmental angiogenesis in the retina, but a 50% reduction in pathological angiogenesis in synovial inflammation (blood vessels in synovial tissue WT 20 ± 5.07 vs. NIK^-/-^ 10.2 ± 3.02).

## Conclusions

NIK is preferentially expressed in EC in RA ST and non-canonical NF-kB signaling in EC results in enhanced angiogenesis in vitro. NIK^-/-^ mice exhibited normal developmental and VEGF-induced angiogenesis, but reduced pathological angiogenesis in AIA. These findings point towards an important role of the non-canonical NF-kB pathway in pathological angiogenesis associated with chronic (synovial) inflammation. This could be exploited for the development of future new therapies for RA.

